# Main findings and advances in bioinformatics and biomedical engineering- IWBBIO 2018

**DOI:** 10.1186/s12859-020-3467-0

**Published:** 2020-05-05

**Authors:** Olga Valenzuela, Fernando Rojas, Ignacio Rojas, Peter Glosekotter

**Affiliations:** 1 Faculty of Sciences, Applied Mathematics, University of Granada, Avenida de Fuente Nueva, Granada, 18071 Spain; 2 Information and Communications Technology Centre (CITIC and ETSIIT-UGR) University of Granada, Periodista Daniel Saucedo Aranda, Granada, 18071 Spain; 3 Department of Electrical Engineering and Computer Science, University of Applied Sciences of Munster, Stegerweldstr 39, Steinfurt, 48565 Germany

**Keywords:** High performance in Bioinformatics, Next generation sequencing and sequence analysis, Biomedicine, Biomedical Engineering, Computational systems for modelling biological processes, Healthcare and diseases

## Abstract

In the current supplement, we are proud to present seventeen relevant contributions from the 6th International Work-Conference on Bioinformatics and Biomedical Engineering (IWBBIO 2018), which was held during April 25-27, 2018 in Granada (Spain). These contributions have been chosen because of their quality and the importance of their findings.

## Introduction. iWBBIO 2018 edition

IWBBIO 2018 Conference seeks to provide a discussion forum for scientists, engineers, educators and students about the latest ideas and realizations in the foundations, theory, models and applications in the field of Bioinformatics and Biomedical Engineering. One of the main objectives of the conference is that research in the bioinformatic field can reach the medical application. The conference sought to focus on diverse fields to create multidisciplinary researches integrating areas like biomedical engineering, computer since, mathematics, artificial intelligence, bioinformatics, statistics or biomedicine [1, 2]. As in previous editions of IWBBIO, it also aims to create a friendly environment that could lead to the establishment of scientific collaborations and exchanges among attendees. These ideas provided important advances to the scientific community in fields like genomics, next-generation sequencing, drug design and advanced pharmacology, biomedical modelling and e-health, among other.

The list of topics in the successive Call for Papers has also evolved, resulting in the following list for the present edition:

1. **Computational proteomics**. Analysis of protein-protein interactions. Protein structure modelling. Analysis of protein functionality. Quantitative proteomics and PTMs. Clinical proteomics. Protein annotation. Data mining in proteomics.
2. **Next generation sequencing and sequence analysis**. De novo sequencing, re-sequencing and assembly. Expression estimation. Alternative splicing discovery. Pathway Analysis. Chip-seq and RNA-Seq analysis. Metagenomics. SNPs prediction.
3. **High performance in Bioinformatics**. Parallelization for biomedical analysis. Biomedical and biological databases. Data mining and biological text processing. Large scale biomedical data integration. Biological and medical ontologies. Novel architecture and technologies (GPU, P2P, Grid,...) for Bioinformatics.
4. **Biomedicine**. Biomedical Computing. Personalized medicine. Nanomedicine. Medical education. Collaborative medicine. Biomedical signal analysis. Biomedicine in industry and society. Electrotherapy and radiotherapy.
5. **Biomedical Engineering**. EComputer-assisted surgery. Therapeutic engineering. Interactive 3D modelling. Clinical engineering. Telemedicine. Biosensors and data acquisition. Intelligent instrumentation. Patient Monitoring. Biomedical robotics. Bio-nanotechnology. Genetic engineering.
6. **Computational systems for modelling biological processes**. Inference of biological networks. Machine learning in Bioinformatics. Classification for biomedical data. Microarray Data Analysis. Simulation and visualization of biological systems. Molecular evolution and phylogenetic modelling.
7. **Healthcare and diseases**. Computational support for clinical decisions. Image visualization and signal analysis. Disease control and diagnosis. Genome-phenome analysis. Biomarker identification. Drug design. Computational immunology.
8. **E-Health**. E-Health technology and devices. E-Health information processing. Telemedicine/E-Health application and services. Medical Image Processing. Video techniques for medical images. Integration of classical medicine and E-Health.

In this edition of IWBBIO, we are honored to have the following invited speaker:

1. **Prof. Joaquin Dopazo**, Fundacion Progreso y Salud, Clinical Bioinformatics Research Area, Sevilla, Spain
2. **Prof. Luis Rueda**, Professor, School of Computer Science, Pattern Recognition and Bioinformatics Lab, Windsor Cancer Research Group, University of Windsor.
3. **Dr. Anagha Joshi**, Bioinformatics Group Leader, Developmental Biology Division, The Roslin Institute, University of Edinburgh, UK
4. **Prof. FangXiang Wu**, P.Eng, SMIEEE Professor, Division of Biomedical Engineering, Professor, Department of Mechanical Engineering, College of Engineering, University of Saskatchewan, 57 Campus Dr., Saskatoon, SK Canada.
5. **Prof. Jiayin Wang**, Professor, Xian Jiaotong University, China.

These plenary lectures strengthened the aim of this conference for the diffusion and the discussion of high quality researches from some of the most recognized scientists in these fields.

During IWBBIO 2018 several Special Sessions will be carried out. Special Sessions will be a very useful tool in order to complement the regular program with new and emerging topics of particular interest for the participating community. Special Sessions that emphasize on multi-disciplinary and transversal aspects, as well as cutting-edge topics are especially encouraged and welcome, and in this edition of IWBBIO 2018 a total of eleven special sessions have been presented

The IWBBIO 2018 has continued as a two-track conference, increasing the number of sessions to a total of 25 oral and 1 poster session. It received more than 210 contributions which were reviewed by at least 3 referees from our estimated program and steering committees. The conference continues accepting both full and abstract submissions for presentations. However, it still maintained a high rate of full contributions against abstracts. IWBBIO 2018 received more than 180 attendees from diverse European nationalities (Spain, United Kingdom, France, Italy, Poland, etc) but also overseas countries like United Stated, Korea, China or India.

## Contributions of this special issue

Those contributions which were considered more relevant taking into account the evaluation and opinion of reviewers and chairmen were then invited to participate in this supplement for the BMC Bioinformatics journal (initially BMC Bioinformatics and BMC Systems Biology were the special issues, but all the papers were merged for a single journal). In the present issue of BMC Bioinformatics journal, it is a pleasure to present you these contributions that provide a clear overview of the thematic areas covered by the IWBBIO conference, ranging from theoretical/review aspects to real-world applications of bioinformatic and biomedical engineering.

The first paper authored by Xin Guan et al. [3], presents a novel method that incorporates domain knowledge in a random forest framework for feature selection (Know-GRRF) which is of great interest at the present time, since the effectiveness of machine learning models can often be dramatically improved by feature selection as a preprocessing step. Besides, domain knowledge incorporation has been widely studied as it can always help to the right selection. As discussed in this paper, in the discovery of biomarkers, the application of domain knowledge is an useful approach to eliminate false positives, prioritize functionally effective markers and facilitate the interpretation of predictive signatures. This article also presents a very interesting application of the proposed (Know-GRRF) to human biomarkers for radiation biodosimetry using non-human primates (NHPs) as experimental subjects. It shows very interesting final results by using Know-GRRF with cross-species correlation as prior knowledge. The authors also built a predictive model gene expression biomarkers, and develop a biodo-simetry model to estimate absorbed dose by a human exposed to radiations in a radiation explosion event.

The articule by Osama Hamzeh et al. [4] has used RNA sequencing data from TCGA to predict tumor locations in prostate cancer tissue using machine learning. The task of finding the tumor location in the prostate is an crucial pathological step for prostate cancer diagnosis and treatment. In fact, authors identified genomic biomarkers for the classification of the locations of prostate cancer. Using SVM-RBF, the classification accuracy was 99%, using a data set that consists of 450 samples, which was the higher than Naive Bayes and Random forest.

There is an important effort of the scientific community in the development of methods to predict new disease genes from protein-protein interaction networks (PPIs). However, PPIs change during the life of the cells (dynamic) and, therefore, only the use of static PPI networks can affect the performance of the algorithms. In the paper presented by Ping Luo et al. [5], the authors propose a disease gene prediction ensemble algorithm based on the centrality characteristics extracted from single-sample PPI clinical networks (EdgCSN). The EdgCSN first builds a network based on a single sample of a PPI network and the clinical expression of the gene of each case sample, and merges it into a network according to the frequency of each edge that occurs in each of the Sample based networks. The next step is the construction of a logistic models, which are trained with centrality features extracted from the fused networks, and an ensemble strategy is used to predict the probability of each gene being disease-associated. The authors have evaluated the proposed methodology, EdgCSN, in several problem: breast cancer (BC), thyroid cancer (TC) and Alzheimer’s disease (AD), obtaining outstanding AUC values of 0.970, 0.971 and 0.966, respectively.

Identification of conserved interactions between proteins and ligands that are reused across a protein family it is a key factor in understanding molecular recognition processes, also facilitating tasks such as the design of effective drug. The advancement in the promotion of computational algorithm to support our understanding of the ligand-receptor recognition process is of fundamental importance in the biological processes. The contribution by Vagner S Ribeiroy et al. [6], presents a method called visGReMLIN, a user-friendly web-server that generates a computational strategy to detect motifs at the protein-ligand interface and a visual interactive platform to investigate and understand such patterns.

Jan Fostier [7] proposed a novel software tool which is able to parallel the algorithm for Position weight matrices (PWMs) matching problems. As presented by the author, the identification of all matches of a large set of PWMs in long DNA sequences requires relevant computational resources. A new algorithm leveraging high performance computing techniques, called BLAMM is presented. The BLAMM algorithm can identify position weight matrix occurrences in DNA sequences, and it can run both in CPUs supporting SIMD instructions and GPUs. The proposed algorithm is efficient and its performance is supported by comprehensive experiments on multiple datasets.

In the contribution by Yixuan Wang et al. [8], authors propose a new algorithm for estimating the distributions of the length of micro-satellites, which are genomic regions that consist of short and repetitive DNA motifs. For any micro-satellite region, it is considered as a micro-satellite instability (MSI) event, if the length distribution sampled from tumor tissue is considerably different from the distribution sampled from the corresponding normal tissue. In this article, the authors propose a probabilistic approach termed ELMSI, which is based on a next generation sequencing approach for MSI testing. The main advantage of the proposed ELMSI is that it is capable of estimating the length on MSIs, which existing software tools fail to do. Experimental of simulated data showed that ELMSI achieved good recall and precision for estimating the length of MSIs.

In the manuscript by Michael G Sadovsky et al. [9], a clustering of DNA triplets of chloroplast genomes is performed. Their clustering shows several natural structures that have different properties to those of other genomic families. The points in 63-dimensional space were clustered due to elastic map technique.Two main observations have been presented: existence of eight clusters, one of which is connected to the presence of non-protein coding genes in choloplasts, and unusual symmetry configuration of the clusters with respect to each other, different from the previously observed in bacterial genomes. Such mirror symmetry yields a separation of the genomes into two groups.

Following, the paper by Sara Nasiri et al. [10] address the challenge of discerning between benign and tumoral skin lesions. This paper was focused on designing a powerful diagnosis tool based on deep learning thanks to the use of convolutional neural networks. Because it is one of the cancer that most affects the population (more common in Caucassian populations, elderly and in developped countries), this is obviously an hot topic to develop the use of both image classification and of text information when disease description and recommendation can be used as images or texts references, which is the context of the proposed methodology presented in this contribution, the so-called DePicT Melanoma Deep-CLASS. The accuracy of the system has been verified by utilizing the ISIC Archive dataset in analysis of skin lesion classification as a benign and malignant melanoma. The kernel of DePicT Melanoma Deep-CLASS is built upon a convolutional neural network (CNN) composed of sixteen layers.

The paper authored by Renzo Angles et al. [11] presents GSP4PDB, a bioinformatics web tool that lets the users design, search and analyze protein-ligand structural patterns inside the Protein Data Bank. GSP4PDB provides a simple graphical interface to draw a graph-based structure pattern and execute search in the system. The contribution describe a protein-ligand structural pattern as a graph such that the nodes represent protein’s components and the edges represent structural relationships and develop a web tool to facilitate its use and adaption.

Following, the paper presented by Hayman Saddik et al. [12] suggests that sarcopenia negatively affects hip bone strength indices in postmenopausal women. As a conclusion, the authors state that implementing strategies to increase skeletal muscle mass index (SMI) in postmenopausal women may be useful for preventing osteoporotic fractures. The study presented in this contribution included 8 postmenopausal women (aged between 65 and 84 years) with sarcopenia and 60 age-matched controls (with normal SMI).

The paper by G.V. Zhikhareva et al. [13] presents a novel methodology to increase the informative value of electrocardiographic (ECG) surveys using data from multichannel electrocardiographic leads placed on the surface of the human torso. This contribution deals with a procedure for computing Body Surface Potential Mapping (BSPM) of the torso and compares it to the method of Reconstruction of Equivalent Electrical Sources on Heart Surface (HSSM). The dynamics of the heart electrical activity is defined by space-time mapping of equivalent electrical sources in HSSM. Inverse calculations are based on the Tikhonov method.

Wenting Wu et al. [14] address in their article a relevant tumor, the cervical cancer, which is the fourth most common tumor in women worldwide, mostly resulting from human papillomavirus (HPV). This study explores the relationships between high-risk human papillomavirus (HR-HPV) and cervical cancer by integrating biological data and mathematical modeling techniques. A total of 16693 patients were studied from July 2016 to July 2017 in the outpatient department of the General Hospital of the People’s Liberation Army. The authors statistically analyzed infection data for 13 HR-HPV types in 4 precancerous stages. The results showed that the overall prevalence rate of the 13 HR-HPV types (16.64%) is less than the previous, but HPV52, HPV58 and HPV16 still have the greatest impact on the health of women in China. By clustering analysis, biological homology results in similar infection rate trends in precancerous stages was found and finally, the single/multiple infection proportions of HR-HPV demonstrated a trend that the multiple infections rates of HR-HPV increased as the disease developed.

The subject of the paper by Ayca Kirimtat et al. [15]- exploring the possibility of using affordable smartphone-based IR cameras for biomedical applications - is of interest to the biomedical community. Since the inhomogeneous body temperature is a relevant indicator of severe injuries, abrasions, and illnesses, infrared thermography is the strongest method among other conventional methods to map the skin temperature variations. In this contribution, the authors presented a biomedical applications that include skin cancer screening, wound detection in a diabetic foot, muscle activation assessment during an exercise, or thermal mapping of healthy human bodies. This paper focuses on analysing temperature distribution on the injured toe of a subject with two different smartphone-based infrared camera models namely FLIR One and SEEK Compact Pro.

The next article, by Ana Cernea et al. [16], is devoted to the actual problem of predicting the phenotype using the modeling of genetic networks that can help to understand the causes of the disease. In this article, the authors compared three new methods. The first algorithm (Fisher’s ratio sampler) selects the most discriminatory genes and samples the high discriminatory genetic networks according to a prior probability that it is proportional to their individual Fisher’s ratio. In the second one, (holdout sampler) is based in the bootstrapping procedure used in regression analysis, to found the most frequently sampled genes. The third one is a pure random sampler which randomly builds networks of differentially expressed genes. The authors use these algorithms to analyze the genetic pathways involved in metastasis and survival in triple negative breast cancer.

Simulation of microfluidic devices for the analysis of blood samples is a great tool for optimizing these devices, requiring a sufficient degree of simulation accuracy. Accuracy is ensured by measuring appropriate values which inform about the course of the simulation and can also be measured in a real experiment. The paper from Hynek Bachraty et al. [17] presents a methodology based on machine learning in which the data we have gained from simulation are used to improve the quality of data processing from video from a real experiment.

In the paper by Annarita Fanizzi et al. [18] an automatic model for characterizing and discriminating tissue in normal/abnormal and benign/malign in digital mammograms, as support tool for the radiologists, is presented. It is important to highlight that an early diagnosis of breast lesions increases the chances of survival and reduce the mortality rate, being this tumor (breast cancer), the most widespread in the female population. The author trained a Random Forest classifier on some textural features extracted on a multiscale image decomposition based on the Haar wavelet transform combined with the interest points and corners detected by using Speeded Up Robust Feature (SURF) and Minimum Eigenvalue Algorithm (MinEigenAlg), respectively. The methodology was tested in 260 ROIs extracted from digital mammograms of the BCDR public database. The model proposed was high performing in the prediction of the normal/abnormal and benign/malignant ROIs.

Finally, Cristina Soguero-Ruiz et al. [19] address in their contribution the analysis of healthy and chronic patients associated with the University Hospital of Fuenlabrada in Spain. The diseases are diabetes mellitus (DM) and essential hypertension (EH) are chronic diseases more prevalent every year, both independently and jointly. The proposed method used decision trees as a tool for selecting discriminative features and making predictive analyses of the health status of this kind of chronic patients.

## Conclusions

The articles presented in this special issue provides insights related to Bioinformatics and Biomedicine Engineering. As Guest editors, we would like to express our thankfulness to all the authors contributing with their high quality researches to the achievement of this supplement. Also, we are very grateful to expert scientists that have actively collaborated with their recommendations and suggestions to review and improve these contributions. We specially thank to Mr. Omar El Bakry for his excellent and constant support with the publication and edition of this supplement. It has been an honor for us to participate in it. We finally invite authors and readers of this supplement to submit their recent works to future editions of IWBBIO, which will be announced at http://iwbbio.ugr.es. We wish the readers can benefit from insights of these relevant papers, and contribute to these rapidly and dynamics growing areas.
